# DW-UNet: Loss Balance under Local-Patch for 3D Infection Segmentation from COVID-19 CT Images

**DOI:** 10.3390/diagnostics11111942

**Published:** 2021-10-20

**Authors:** Cheng Chen, Jiancang Zhou, Kangneng Zhou, Zhiliang Wang, Ruoxiu Xiao

**Affiliations:** 1 School of Computer and Communication Engineering, University of Science and Technology Beijing, Beijing 100083, China; b20170310@xs.ustb.edu.cn (C.C.); elliszkn@163.com (K.Z.); wzl@ustb.edu.cn (Z.W.); 2 Department of Critical Care Medicine, Sir Run Run Shaw Hospital, Zhejiang University School of Medicine, Hangzhou 310016, China; jiancangzhou@zju.edu.cn

**Keywords:** COVID-19, infection segmentation, 3D convolutional neural network, data enhancement, weighted loss function

## Abstract

(1) Background: COVID-19 has been global epidemic. This work aims to extract 3D infection from COVID-19 CT images; (2) Methods: Firstly, COVID-19 CT images are processed with lung region extraction and data enhancement. In this strategy, gradient changes of voxels in different directions respond to geometric characteristics. Due to the complexity of tubular tissues in lung region, they are clustered to the lung parenchyma center based on their filtered possibility. Thus, infection is improved after data enhancement. Then, deep weighted UNet is established to refining 3D infection texture, and weighted loss function is introduced. It changes cost calculation of different samples, causing target samples to dominate convergence direction. Finally, the trained network effectively extracts 3D infection from CT images by adjusting driving strategy of different samples. (3) Results: Using Accuracy, Precision, Recall and Coincidence rate, 20 subjects from a private dataset and eight subjects from Kaggle Competition COVID-19 CT dataset tested this method in hold-out validation framework. This work achieved good performance both in the private dataset (99.94–00.02%, 60.42–11.25%, 70.79–09.35% and 63.15–08.35%) and public dataset (99.73–00.12%, 77.02–06.06%, 41.23–08.61% and 52.50–08.18%). We also applied some extra indicators to test data augmentation and different models. The statistical tests have verified the significant difference of different models. (4) Conclusions: This study provides a COVID-19 infection segmentation technology, which provides an important prerequisite for the quantitative analysis of COVID-19 CT images.

## 1. Introduction

COVID-19 is currently the most serious infectious lung disease [1,2]. Therefore, clinical imaging examination is a common and critical process. Usually, professional levels and subjective judgments of different doctors leads to inconsistent diagnosis results [3,4,5]. Although clinical medical technology has a set of quantitative indicators for preliminary screening [6,7,8,9], it is very dependent on collection and quantification of image information, which limits promotion of this technology [10,11]. Thus, reconstruction of COVID-19 infection can provide a digital model and quantitative analysis basis for diagnosis and treatment of COVID-19. It helps to build a more objective evaluation system and provides theoretical guidance for staff in related fields [12,13,14].

**Figure 1:**
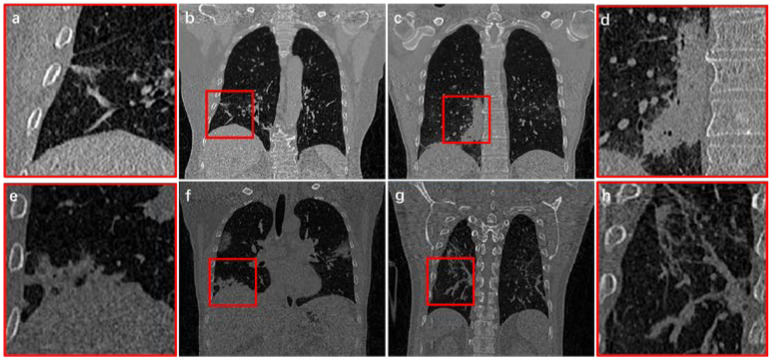
Infection structure from COVID-19 CT images. (**b**,**c**,**f**,**g**) indicate four different cases. (**a**,**d**,**e**,**h**) are the local magnification structure of the red boxes from (**b**,**c**,**f**,**g**), respectively.

Since CT images can collect 3D information of patients, it is currently the most commonly clinical imaging technique for COVID-19 examination [15,16,17,18]. From CT images, COVID-19 infection present characteristics of regional spread, blurred boundaries, tissue adhesion, and large morphological differences (Figure 1). Therefore, identifying the method of how to extract infection effectively and accurately from COVID-19 CT images is a difficult and urgent challenge [19,20].

To solve this problem, many researchers have explored it. For examples, Fan et al. [21] proposed a deep network for lung infection segmentation to automatically identify COVID-19 infected area in CT images. In their work, a semi-supervised framework based on random propagation is applied to make up for the lack of COVID-19 CT data. Qiu et al. [22] introduced a lightweight model. Compared with classic network structure, their parameters have been significantly reduced, which can meet needs of rapid clinical training. Yan et al. [23] established a global energy network to analyze COVID-19 CT images. Through their network, global energy was increased, thereby enhancing contrast of infection. Also, a feature mutation module has been added to adaptively adjust global attributes applied as analysis of COVID-19 features. Chen et al. [24] proposed a 3D attention module to enhance infection feature from COVID-19 CT images. Their analysis of the pros and cons are listed in Table 1.

**Figure 2:**
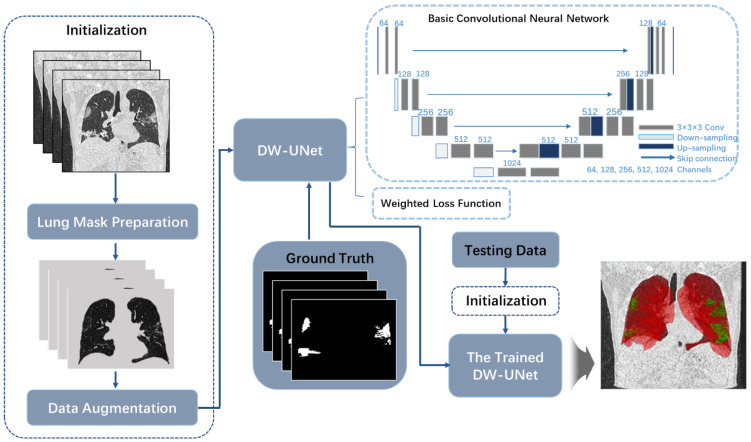
Method flow chart in this work.

Although initial results have been achieved in reconstruction of COVID-19 infection, there are still some problems that still need to be solved: (1) When infections are adjacent to thorax, segmentation boundary cannot be effectively judged (Figure 1c); (2) Most methods focus on 2D images processing or partial patches, which cannot deal with complete 3D feature information in CT images; (3) COVID-19 infection usually only occupies a small part of the entire CT image. Thus, imbalance of positive and negative samples appears in network training, resulting in slow convergence and insufficient accuracy; (4) In vicinity of infected area, invasive structure of infection adheres to vessels and tracheas, which is difficult to distinguish effectively (Figure 1d).

In order to solve the above existing problems, this work proposes a method for analyzing COVID-19 CT images to meet the needs of reconstruction and quantification of infection. The main contributions are as follows: (1) 3D COVID-19 infection from CT images can effectively constructed by proposed deep weighted UNet (DW-UNet), which has 3d structure and space information; (2) The proposed data enhancement method can reduce interference of vessels and trachea to infection, which improves segmentation contrast between target and background; (3) An application example of weighted loss function in 3D network segmentation is introduced, which provides a theoretical basis for expanding to other fields. The main structure of this work is: The second section elaborates on the method described. The third section introduces the experimental process and conclusion analysis. Finally, the work is summarized in the fourth section.

## 2. Methods

This work proposes a method that can identify infection and complete segmentation from COVID-19 CT image. The main steps include: Firstly, the infected lung masks are prepared based on Random Walker segmentation. By these masks, the area of image processing can be focused on the lung region. Secondly, based on tubular feature of vessels and tracheas in 3D voxels, data enhancement is achieved. Finally, DW-UNet based on weighted loss function is established, which can balance convergence speed of different samples and improve segmentation accuracy. Method flow chart is shown in Figure 2.

### 2.1. Lung Mask Preparation

Lung mask preparation is mainly used to extract lung area that only contains lung tissue, tracheas, vessels, and infection. Sometimes, infection is located at lungs borders adhere to tissues, resulting in blurring and rupture of the lung verge. Therefore, Random Walker method [25] is adopted to complete pathological lung extraction. Firstly, structured data are established and defined as V=(P,E) based on lung CT images. Where *P* and *E* represent sets of voxel points and edges between voxels, which satisfies p∈P and e∈E⊆P×P. To measure degree of similarity between points, edge weights based on gray and space are defined as follows:

(1) $$w_{ij}=exp\left(-\beta_{1}{\left(g_{i}-g_{j}\right)}^{2}-\beta_{2}{\left(d_{i}-d_{j}\right)}^{2}\right)$$

where $\beta_{1}$ and $\beta_{2}$ parameters control influence of different factors. *g* and *d* are gray value and spatial index of voxel point *p*.

By edge weights, probability of a non-marked point walking to each seed point can be calculated. Then, the maximum probability can be applied as new mark of the non-marked point to achieve image segmentation. Reference [26] proved that solution of probabilities can be transformed into a classic Dirichlet problem: By finding a harmonic function, it is solution of a specified partial differential equation in a given area, and its predetermined value is taken on this area boundary. Since harmonic function satisfies Laplace equation $\nabla^{2}u=0$ and corresponds to the Euler–Lagrange equation of Dirichlet integral D[u], so the solution when Dirichlet integral reaches the minimum is harmonic function. Additionally, $D\left[u\right]=\frac{1}{2}\int_{\Omega }{\left|\nabla u\right|}^{2}d\Omega$.

Laplacian matrix *L* of *V* is established as:

(2) $$L_{ij}=\left\{ \begin{array}{ll} d_{i} & ,\;when\;i=j\\ -w_{ij} & ,\;when\;v_{i}\;and\;v_{j}\;are\;adjacent\\ 0 & ,\;others \end{array}\right.$$

**Figure 3:**
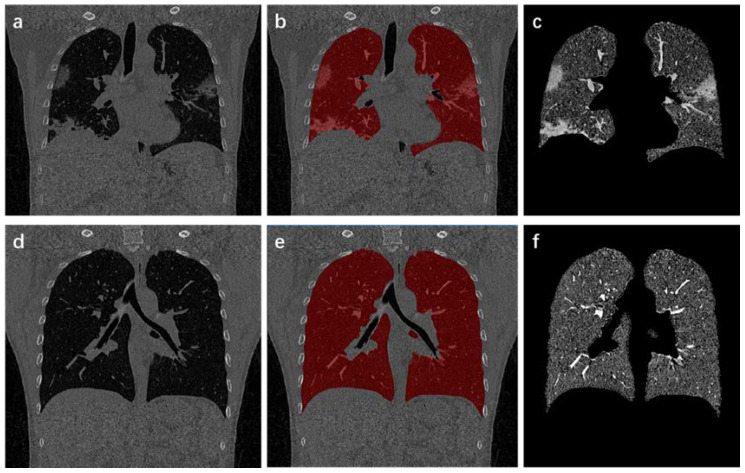
Process and results of lung region extraction. (**a**,**d**) are the original CT images; (**b**,**e**) show results of lung mask covering the original CT images; (**c**,**f**) indicate the extracted lung area.

*L* satisfies $L=A^{T}CA$; $d_{i}$ is the degree of vertex $v_{i}$, which is sum of weights of all edges connecting vertex. It satisfies $d_{i}=\sum w_{ij}$. Vertex *P* is composed of marked points $U_{s}$ and non-marked points $U_{u}$, satisfying $U_{u}\cup U_{s}=V$ and $U_{u}\cap U_{s}=Ø$. Thus, discrete expression D[x] is defined as:

(3) $$\begin{array}{ll} D\left[x_{u}\right] & =\frac{1}{2}\left[x_{s}^{T}x_{u}^{T}\right]\left[\begin{array}{cc} L_{M} & B\\ B^{T} & L_{U} \end{array}\right]\left[\begin{array}{l} x_{s}\\ x_{u} \end{array}\right]\\ & =\frac{1}{2}\left(x_{s}^{T}L_{u}x_{s}+2x_{u}^{T}B^{T}x_{s}+x_{u}^{T}L_{u}x_{u}\right) \end{array}$$

where $x_{s}$ and $x_{u}$ correspond to probabilities of marked and non-marked points, respectively. To obtain $x_{u}$, differential of $D\left[x_{u}\right]$ with respect to $x_{u}$ is solved, and the extreme point through the zero point is found. $L_{u}x_{u}=-B^{T}x_{s}$ is satisfied after solving. $x_{i}^{s}$ is supposed to represent probability that vertex $v_{i}$ belongs to label *s*. Set of *s* tags is defined as $Q\left(v_{j}\right)=s$ and $\forall v_{j}\in U_{s}$, where 0<s≤K and *K* is the number of all seed points. Thus, solution of Dirichlet problem is $L_{U}X=-B^{T}M$. Segmentation result is obtained through this equation (Figure 3). For any vertex, the sum of all probabilities is 1. So $\sum_{s}x_{i}^{s}=1,\forall v_{i}\in V$ is satisfied.

### 2.2. Data Enhancement

**Figure 4:**
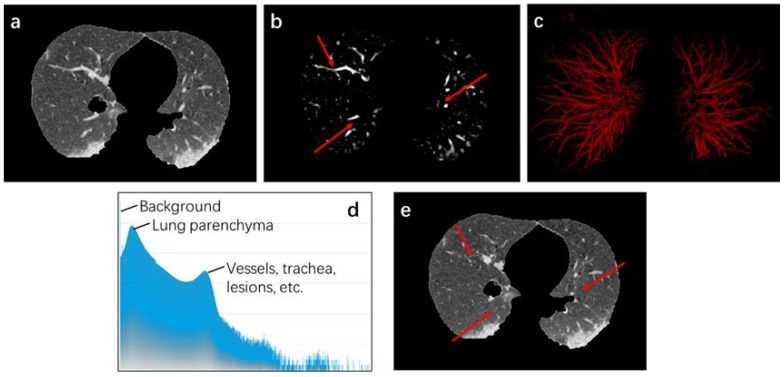
Data Enhancement process. (**a**) represents the original data; (**b**) shows the enhanced data; for the convenience of observation, (**c**) is segmentation result under 0.8 threshold; (**d**) reveals gray histogram of original data; (**e**) is result after data enhancement. The area pointed by the red arrows in (**b**,**e**) was suppressed after data enhancement.

Figure 4 reveals the data enhancement process. In the extracted lung area structure, it can be observed that the contrast between infection and vessels or tracheas is low. To eliminate interference of these tissues, contrast of infection is improved through data enhancement. Thus, to extract structure information of COVID-19 CT images, Hessian matrix of 3D medical image I(x,y,z) is constructed and expressed as:

(4) $$H=\nabla^{2}I=\left(\begin{array}{ccc} I_{xx} & I_{xy} & I_{xz}\\ I_{yx} & I_{yy} & I_{yz}\\ I_{zx} & I_{zy} & I_{zz} \end{array}\right)$$

where $I_{xx}$, $I_{xy}$, and $I_{xz}$ correspond to the second-order partial differential of I(x,y,z), respectively. In digital images, the second-order partial differentials in *X*, *Y*, and *Z* directions are expressed in a discrete manner:

(5) $$\left\{ \begin{array}{l} I_{xx}=\frac{\partial^{2}I}{\partial x^{2}}=I\left(x-1,y,z\right)+I\left(x+1,y,z\right)-2I\left(x,y,z\right)\\ I_{yy}=\frac{\partial^{2}I}{\partial y^{2}}=I\left(x,y-1,z\right)+I\left(x,y+1,z\right)-2I\left(x,y,z\right)\\ I_{zz}=\frac{\partial^{2}I}{\partial z^{2}}=I\left(x,y,z-1\right)+I\left(x,y,z+1\right)-2I\left(x,y,z\right) \end{array}\right.$$

The corresponding mixed partial differential can be expressed as:

(6) $$\left\{ \begin{array}{ll} I_{xy}=I_{yx}=\frac{\partial^{2}I}{\partial x\partial y} & =I\left(x+1,y+1,z\right)+I\left(x,y,z\right)\\ & -I\left(x+1,y,z\right)-I\left(x,y+1,z\right)\\ I_{yz}=I_{zy}=\frac{\partial^{2}I}{\partial y\partial z} & =I\left(x,y+1,z+1\right)+I\left(x,y,z\right)\\ & -I\left(x,y+1,z\right)-I\left(x,y,z+1\right)\\ I_{xz}=I_{zx}=\frac{\partial^{2}I}{\partial x\partial z} & =I\left(x+1,y,z+1\right)+I\left(x,y,z\right)\\ & -I\left(x+1,y,z\right)-I\left(x,y,z+1\right) \end{array}\right.$$

Since Hessian matrix is a symmetric matrix, eigenvalues $\lambda_{1}$, $\lambda_{2}$, and $\lambda_{3}$$\left(\left|\lambda_{1}\right|\leq \left|\lambda_{2}\right|\leq \left|\lambda_{3}\right|\right)$ can be obtained and the corresponding eigenvectors are defined as $\vec{v_{1}}$, $\vec{v_{1}}$, and $\vec{v_{3}}$. Based on its geometric meaning, $\lambda_{1}$ represents intensity change in direction of vessels and tracheas ($\vec{v_{1}}$). $\lambda_{2}$ and $\lambda_{3}$ represent intensity change in direction perpendicular to vessels and tracheas ($\vec{v_{2}}$ and $\vec{v_{3}}$).

In CT images, according to vessels and tracheas characteristic, they always present a bright tubular structure in contrast to a relatively dark background. Usually, intensity change along the main direction of them is much smaller than intensity change along their vertical direction. Based on this prior knowledge, it can be used to distinguish tube and other structures in image. Therefore, when a pixel with larger $\lambda_{2}$ and $\lambda_{3}$ obtains a smaller $\lambda_{1}$, it has a greater probability of belonging to vessels or tracheas. Inspired by [27], construction of a linear filter based on 3D voxels is as follows:

(7) $$H_{\lambda s}(x)=\left\{ \begin{array}{l} 0\;\lambda_{2}>0\;\mathrm{or}\;\lambda_{3}>0\\ (1-\exp(-\frac{R_{A}^{2}}{2\alpha^{2}}))\exp(-\frac{R_{B}^{2}}{2\beta^{2}})(1-\exp(-\frac{S^{2}}{2c^{2}}))\;\mathrm{else} \end{array}\right.$$

where *x* is voxel point in volume data; α, β, and c control the difference between tubular and disc structure, tubular and spherical structure, and high-contrast and low-contrast structure, respectively. Therefore, by adjusting the three control parameters, tubular structure can be effectively enhanced. To augment data, new voxel I′(x,y,z) is updated as follows:

(8) $$I\prime (x,y,z)=\left\{ \begin{array}{ll} T & H_{\lambda s}(x)\geq 0.8\\ I(x,y,z) & H_{\lambda s}(x)<0.8 \end{array}\right.$$

where *T* is obtained from gray distribution H(I) of I(x,y,z), which includes two cluster centers. The largest cluster center usually has a low gray value and is mainly composed of lung tissue with low brightness. Frequency of another cluster center is low, mainly composed of highlighted vessels, tracheas, and infected areas. Thus, *T* is generated randomly from lung tissue cluster. By processing, the obvious vessels and tracheas in original data can be effectively suppressed and impact on infection can be minimized as much as possible.

### 2.3. Structure of Basic Convolutional Neural Network

Deep weighted UNet (DW-UNet) is combined with basic convolutional neural network and weighted loss function. Since 3D U-Net [28] has advantages of lightweight and symmetry in 3D-based networks, this work established it as basic convolutional neural network. Its main structure is an encoder-decoder way, in which the first half contains encoding path used for feature extraction, and the second half is applied for decoding path of feature analysis and pixel classification. In the encoding path, there are five resolution layers. Each layer has two convolutional layers with 3 × 3 × 3 kernel size, and a batch normalization step is performed to obtain consistent data distribution. Then, ReLU function is applied to complete activation. A 2 × 2 × 2 maximum pooling operation with a 2 stride is adopted to realize down-sampling. In addition, the number of feature channels along each layer of encoding path is doubled in turn.

In the decoding path, structure is symmetrical with encoding path to ensure that the final structure matches input size. Up-sampling with a 2 × 2 × 2 kernel size is adopted, and step is 2. Two convolutions are also 3 × 3 × 3 kernel size. Then, normalization and ReLU steps are completed. The number of feature channels is sequentially halved along each successive layer in decoding path. The last convolution is used to map final component feature vector to a single channel and Sigmoid activation function is applied for classification. In addition, in each layer with the same resolution, a skip connection is constructed by passing corresponding feature map from encoding path to decoding path to fuse shallow and deep features.

### 2.4. Weighted Loss Function

Usually, even if independent lung structures have been extracted through lung masks, COVID-19 infection still only occupy a small part. Therefore, convolutional neural network for feature extraction from COVID-19 CT images faces problem of uneven distribution of positive and negative samples. Common loss functions generally keep the same cost for positive and negative samples, resulting in the final convergence of network being dominated by negative samples, and it is impossible to extract effective positive sample feature structures. To introduce weights of positive and negative samples, [29] et al. proposed focal loss function $L_{f}$. In their work, y∈{0,1} is supposed as the sample category, which is background area and lesion area.

p∈[0,1] is the predicted value of y=1 by the model, so that $p_{t}$ satisfies:

(9) $$p_{t}=\left\{ \begin{array}{ll} p & \;\mathrm{if}\;y=1\\ 1-p & \;\mathrm{else} \end{array}\right.$$

Weight factor α is defined to represent proportional weight distribution of positive and negative samples, satisfying:

(10) $$\alpha_{t}=\left\{ \begin{array}{ll} \alpha & \;\mathrm{if}\;y=1\\ 1-\alpha & \;\mathrm{else} \end{array}\right.$$

Then $L_{f}\left(\alpha ,\gamma \right)$ is defined as follows:

(11) $$L_{f}\left(\alpha ,\gamma \right)=-\alpha_{t}{\left(1-p_{t}\right)}^{\gamma }\log\left(p_{t}\right)$$

where γ balances weight distribution of simple and complex samples.

Although $L_{f}\left(\alpha ,\gamma \right)$ changes weights by introducing α and γ factors, the overall cost is also reduced because of weight coefficient. It makes loss value decrease rapidly, resulting in network converging too fast.

To obtain a more reliable loss value and change weight of positive, negative, simple, and complex samples, this work proposes $L_{w}\left(\alpha ,\gamma \right)$, which defines as follows:

(12) $$L_{w}\left(\alpha ,\gamma \right)=-\left(\alpha +1\right)y{\left(2-p\right)}^{\gamma }\log\left(p\right)-\left(1-\alpha \right)\left(1-y\right){\left(1+p\right)}^{\gamma }\log\left(1-p\right)$$

Weighted loss function considers weight distribution of positive, negative, simple, and complex samples. While increasing cost ratio of positive samples, the overall sample cost stays steady, so that the final loss value can maintain overall balance.

## 3. Results

### 3.1. Data Source

In assessment process, this work selected two datasets to test the proposed method. In total, 20 CT scan imaging data of patients with confirmed COVID-19 from the Fifth Medical Center of the PLA General Hospital were retrospectively analyzed as the private dataset. Scanning equipment was a high-resolution LightSpeed VCT CT64 scanner (GE MEDICAL SYSTEMS), and scanning range was uniform from entrance of chest cavity to level of posterior intercostal angle. Where, scan parameters were 120 kV voltage, automatic milliamp technology (40–250 mA), noise index (NI) 25, pitch 0.984:1, matrix 512 × 512, slice thickness 5 mm, and a total of 10232 slices. A total of 12 cases were randomly selected as training data, and the remaining eight were testing data. Slices and allocation details of them are reported in Table S1 of the supporting information. In addition, scope of lung infection was discussed by three experienced clinical experts. After unanimous agreement, identification and labeling of infection area was completed as the manual ground truth for assessment. Private dataset is mainly for discussion of ablation experiments.

Another dataset was selected from public COVID-19 dataset of Kaggle, which consisted of COVID-19 CT images provided by Coronavirus [30] and Radiopaedia [31]. Due to insufficient resolution of some data, eight sets of cases were finally taken as testing data to assess performance of network trained under the private dataset. All data consisted of 512 × 512 matrices with a total of 2111 slices, and were labeled by [32] et al. as the manual ground truth. Slices and allocation details of them are reported in Table S2 of the supporting information. Public dataset is used for performance testing to verify the generalization of the method proposed in this work.

### 3.2. Initialization

This work designed three sets of controlled experiments to verify rationality of the proposed method. The first set of experiments explored effectiveness of data enhancement. They compared results obtained with data enhancement and without data enhancement based on U-Net network and binary cross-entropy loss function $L_{bce}$ [33]. The second group of experiments discusses impact of different loss functions. Experimental groups included binary cross-entropy loss function, focal loss function, and weighted loss function. Where, parameters of weighted loss function have been discussed. The third set of experiments explored performance of this method. So, V-Net [34], U-Net, Dense-Net [35], and DenseVoxel-Net [36] networks were established as comparative experiments. Since proportion of COVID-19 infection was too small, these networks uniformly applied extracted lung regions as dataset to improve their performance.

**Figure 5:**
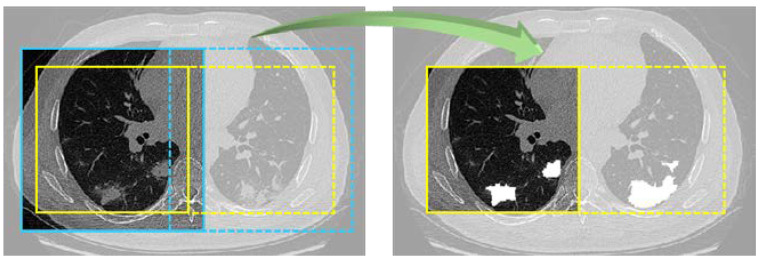
Overlap-tile strategy. Blue line area is output area. Through the instructions of the green 3D arrow, the actual reserved output is yellow line area.

In initialization stage, experiment parameters were set as: small batch of 12 samples, Adam optimizer [37], batch normalization, deep supervision [38], and 100 epochs. To prevent memory congestion, data were randomly cropped into slices with a size of 128 × 128 × 128 in each step, and the current batch of data were read only. In testing progress, testing data were cyclically cropped until the entire data were traversed, and finally the complete splicing of data was realized. In addition, the overlap-tile [39] strategy was adopted: by intercepting yellow line area as actual segmentation result (Figure 5), possible splicing errors were explicitly ignored. The segmentation threshold was set as 0.5.

For quantitative comparison, TP, TN, FP, and FN parameters are set to compare with the manual ground truth. They represent true cases, false positive cases, false negative cases, and true negative cases, respectively, which indicate relationship between predicted value and the manual ground truth. Based on them, Accuracy (ACC), Precision (PRE), Recall (REC), and Dice Coefficient (DSC) [40] are defined as assessment indicators:

(13) $$\left\{ \begin{array}{l} ACC=\frac{TP+TN}{TP+TN+FP+FN}\\ PRE=\frac{TP}{TP+FP}\\ REC=\frac{TP}{TP+FN}\\ DSC=\frac{2TP}{2TP+FN+FP} \end{array}\right.$$

where ACC represents proportion of all voxels with correct predictions, including TP and TN; PRE introduces accuracy of the predicted positive cases; REC is the accuracy of correct infection prediction; DSC reveals coincidence rate of predicted value and the manual ground truth.

Based on these metrics, all experimental results were recorded in Table 2, Table 3, Table 4, Table 5, Table 6, Table 7, Table 8 and Table 9 with CI 95% indices. To better observe the performance, SPE [41], MCC [42], and Kappa [43] indicators have been added to the results of data augmentation (Table 2) and performance assessment (Table 7). Ablation experiments and performance assessments are discussed in next section.

## 4. Discussion

### 4.1. Ablation Experiment

Some ablation experiments were designed to assess effectiveness of data enhancement and weighted loss function in this work. To test effect of data enhancement, all data were extracted with lung mask as original dataset and binary cross-entropy loss function was applied. One set of experiments used original data for training and testing. Data of another experiment were calculated with data enhancement as enhanced dataset. Experiment parameters of two experiments remained the same. By comparison, quantitative results are revealed in Table 2. Based on ACC and PRE, both experiments obtained a high value. Even PRE obtained from original data is 15% higher than that from enhanced dataset. However, REC obtained from original data is only 26.86%. It can be judged that texture characteristics of COVID-19 infection are suppressed due to interference of tissue in original data. Outputs obtained through network usually get fewer positive cases at infection. Although most of these positive cases belong to correct positive cases, they cannot effectively describe overall infection profile. After data enhancement, infection features are enhanced, so that more infection areas are judged as positive cases. Therefore, this is also the reason why DSC obtained by original data is only 37.5%, which is much lower than that of enhanced data.

Since the DSC is the coincidence rate of the segmentation results and the manual ground truth, it is a key indicator that reflects the performance of the segmentation method. Based it, all data pixels are used as samples, and McNemar’s test [44] have verified the results of data augmentation (Table 3). Var.1 indicates whether augmentation is used, and Var.2 indicates whether the voxels overlap. Since *p* = sig. is much less than 0.01, the difference between the two methods is very significant. Therefore, the data augmentation can improve the performance. Details of the Var.1 and Var.2 crosstabulation is shown in Table S3 of supporting information.

**Figure 6:**
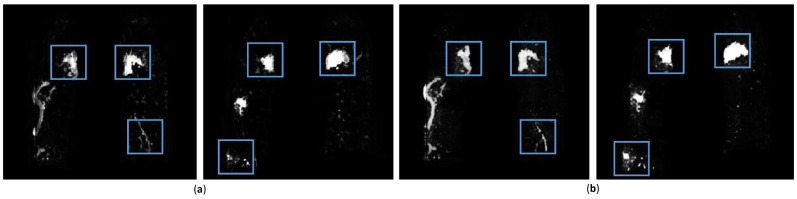
Two output maps from network. (**a**) is results trained by original data. (**b**) is results trained by enhanced data.

**Figure 7:**
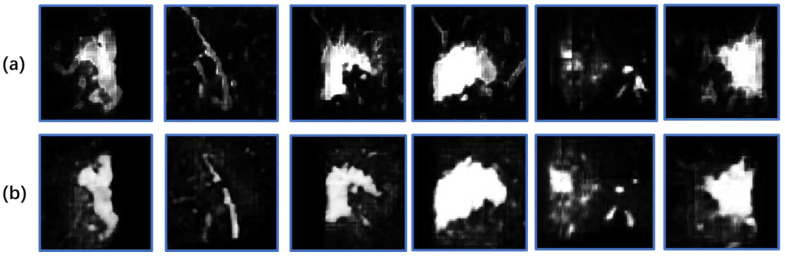
Map features from COVID-19 infection. (**a**) is results obtained without data enhancement. (**b**) is results obtained with data enhancement.

To verify quantitative conclusion, Figure 6 randomly shows two visualizations obtained from enhanced data and original data. Where, Figure 6a shows output map from network trained by original data. Relevant features are marked by blue boxed to highlight. Figure 6b is output map by enhanced data. For convenience of observation, Figure 7 enlarges blue boxes in Figure 6 to display clearly. From visualization results, number of positive cases in infection area from original data is less than that from enhanced data. Additionally, the probability of many areas is lower. This is because infection feature is not obvious which leads to unclear judgment of network. It may lead to wrong judgment as background area in next binary segmentation process, thereby reducing accuracy. However, results from data enhancement also have shortcomings. For example, parts of the invasive lesions are judged to be highly similar vascular structures (Figure 7b, the second column), which may reduce infection area. Therefore, more scientific data enhancement methods are still an important direction for our next exploration.

Another ablation experiment was applied to verify the role of the loss function in the method in this work. In experimental group and control group, lung region extraction, data enhancement, and network parameters were consistent. Additionally, modulation factors α and γ were discussed.

Table 4, Table 5 and Table 6 list quantitative results by focal loss function, weighted loss function, and comprehensive comparison, respectively. For focal loss function, costs of positive and negative samples are balanced by α parameters. When α∈[0,1], cost of positive samples is reduced to α times than the original, and the cost of negative samples is reduced to (1−α) times than the original. In contrast, when α>0.5, positive samples can get more cost, so that network has more time to optimize positive sample. Therefore, Table 4 shows that the best results are usually obtained at α=0.8. γ parameter is mainly used to balance cost of complex and simple samples. Here situation of γ=1 and γ=2 are discussed. By quantitative assessment, performance of γ=2 is better and stable.

Table 5 mainly discuss the parameters of weighted loss function. Different from focal loss function, weighted loss increases cost of positive samples by (1+α) times with α∈[0,1], while cost of negative samples is reduced by (1−α) times. This allows positive samples to get more cost ratio and keep all cost steady, which makes network converge stably while pay more attention to target area. If parameters are appropriate, cost of negative samples will not decrease too much, which prevents network from converging too fast and oscillating near the minimum point. From experimental results, α=0.8 is a better balance. At the same time, due to the influence of γ parameter on cost, weighted loss function has a relatively reliable result at γ=1.

**Figure 8:**
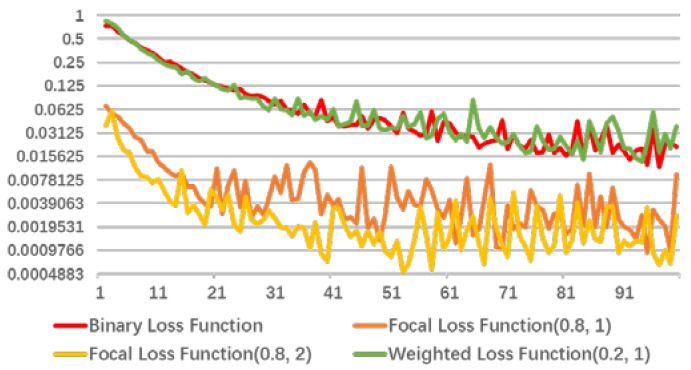
Loss convergence of binary cross-entropy loss function, focal loss (0.8, 1), focal loss (0.9, 2) and weighted loss (0.2, 1) in the first 100 steps.

**Figure 9:**
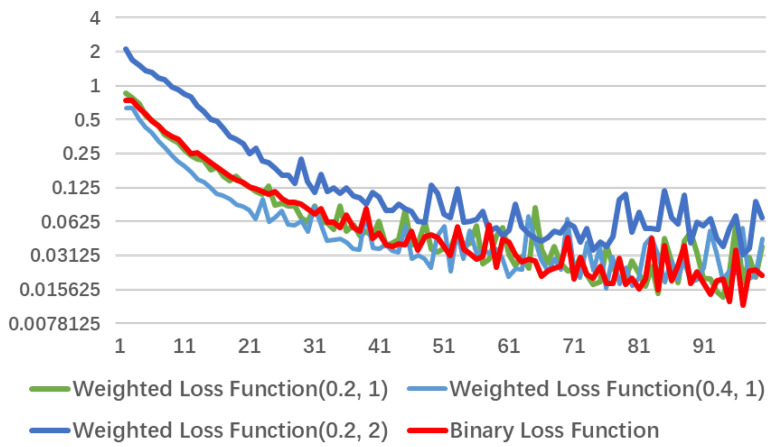
Loss convergence of binary cross-entropy loss function, weighted loss (0.4, 1), focal loss (0.2, 2), and weighted loss (0.2, 1) in the first 100 steps.

Figure 8 and Figure 9 shows loss convergence of three loss functions in the first 100 steps. For convenience of observation, the ordinate adopts a logarithmic coordinate, and base is 2. Where, Figure 8 highlights the relationship between focal loss function and binary cross-entropy loss function. It can be clearly observed that cost of focal loss function drops fast and quickly reaches a relatively stable level. Further, as weight of positive sample increases and weight of the negative sample decreases, trend of this cost reduction will become greater. When facing a sample with a small proportion of positive samples, the weight of the positive and negative samples and the overall cost need to be rebalanced. Figure 9 is the relationship between weighted loss function and binary cross-entropy loss function, where weighted loss function will increase weight ratio of the positive sample to the negative sample while α increases. Similarly, as the number of positive samples is small, it causes overall cost to gradually decrease. Eventually, it will face the same contradiction as focal loss function, so α value needs to be adjusted appropriately. In addition, the overall loss will increase to cause more training cost with γ=2. After discussion of this work, appropriate results are obtained for α=0.2 and γ=1.

**Figure 10:**
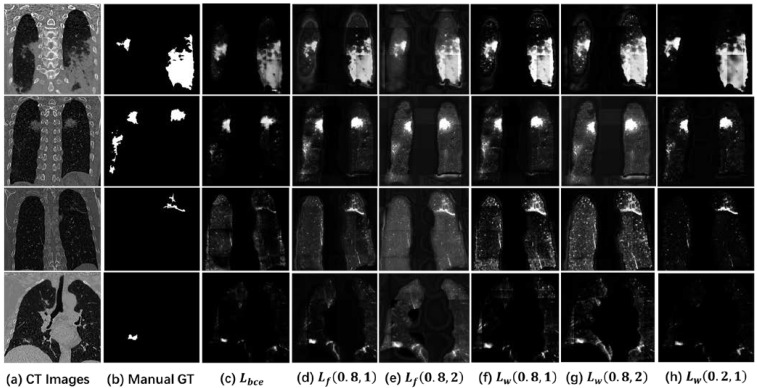
Output maps of four randomly data. (**a**) is original CT images. (**b**) is the manual ground truth. (**c**) is results using binary cross-entropy loss function. (**d**) is results using focal loss (0.8, 1). (**e**) is results using focal loss (0.8, 2). (**f**) is results using weighted loss (0.8, 1). (**g**) is results using weighted loss (0.8, 2). And (**h**) is results using weighted loss (0.2, 1).

To comprehensively discuss binary cross-entropy loss function, focal loss function, and weighted loss function, Table 6 compares results with representative parameters and binary cross-entropy loss function. Comparison shows that on the basis of focal loss function, by changing cost of positive, negative, complex and simple samples, performance can be effectively improved, especially on data where positive and negative samples do not match completely. Therefore, segmentation of vessels, tumors, nodules, and other small tissues are also the same common problem as this work. However, focal loss function will reduce cost of all samples, causing cost to quickly decrease to a small value, which may lead network to be difficult to continue to effectively converge in subsequent training process. Therefore, weighted loss function recalculates cost calculation method for positive, negative, complex, and simple samples, thereby further improving result on the basis of focal loss function. Figure 10 introduces output map of four randomly data. Starting from Figure 10a to Figure 10h, they are original CT images, the manual ground truth, results of binary cross-entropy loss function, focal loss (0.8, 1), focal loss (0.8, 2), weighted loss (0.8, 1), weighted loss (0.8, 2), and weighted loss (0.2, 1). In visualization results, the positive sample area from binary cross-entropy loss function is dark. It can be judged that the same cost weight is difficult to effectively increase output value of target area on data with positive and negative sample imbalance. It is because the cost change obtained by calculating the positive sample is much smaller than the cost obtained by calculating the negative sample.

Other visualization results increase ratio of positive sample cost to a certain extent under adjustment of weight, which makes their positive sample area is brighter than that from binary cross-entropy loss function. It has more advantages in the next binary segmentation. The main difference between them is the degree of calculation for negative samples. For example, because rapid decrease in cost reduces subsequent training efficiency, background from focal loss function (Figure 10d,g) is relatively gray. In Figure 10g, background area is also gray. This is because the number of negative samples is large. Increasing γ value will make cost of negative sample grow faster than positive sample, causing them to be unbalanced again. Therefore, it is very important to choose appropriate parameters.

### 4.2. Performance Assessment

This work evaluated accuracy of the proposed method by comparing with different work. Results of this work were obtained from 3D U-Net network model guided by lung region extraction, data enhancement, and weight loss function. Two datasets tested this work. As comparison groups, V-Net, U-Net, Dense-Net, and DenseVoxel-Net as four common 3D networks were constructed. To improve their performance, all data in comparison groups are calculated with lung region extraction and data enhancement. Finally, quantitative comparison on private dataset and public dataset is introduced in Table 7 and Table 9, respectively. It can be seen from comparison that results on private dataset are better than results on public dataset. This is because labeling specifications of different datasets are different. As the surrounding area of COVID-19 infection is usually spread and blurred, it is impossible to effectively distinguish a clear boundary. Thus, labeling on private dataset is mainly based on conservative outlines, with the purpose of accurately identifying and locating COVID-19 infection. Contrastingly, labeling of public dataset is mainly based on accurate coverage of COVID-19 infection. Label area needs to cover all infection ranges, so it usually crosses actual infection boundary. This leads to reason that PRE on public dataset is significantly higher than that on private dataset, but REC is smaller than the private dataset. It proves that positive cases of outputs are mainly concentrated in target area.

Moreover, we conducted statistical tests on the results in Table 7. We have carried out Pearson Chi-Square, Likelihood ratio, Linear-by-Linear, and Association [44] based on the DSC. So, the Chi-Square tests for different models are shown in Table 8. The *p*-value of all statistical tests is far less than 0.01, so there is a significant statistical difference. Details of the Var.1 and Var.2 crosstabulation is shown in Table S4 of supporting information.

In addition to the indicators mentioned above, calibration is also an important factor in evaluating network models [45,46,47]. Different from the machine learning classification problem, the image segmentation task is a pixel-level processing. Inspired by [48], we define expected calibrated error (ECE) as follows:

(14) $$ECE=\sum_{m}^{M}\frac{\left|B_{m}\right|}{n}\left|PRE\left(B_{m}\right)-conf\left(B_{m}\right)\right|$$

where $PRE\left(B_{m}\right)$ represents the average PRE value in a $B_{m}$. $conf\left(B_{m}\right)$ represents the average of all probability values under this $B_{m}$. $B_{m}$ is the interval divided by the probability value. This study divides five $B_{m}$ with a 0.2 gap. Different from [48], because the positive and negative samples are unbalanced, any classifier can get a higher ACC value. Therefore, this study replaces the PRE that judges the positive class as the observation value. So, the ECE is implemented in Table 7. From their ECE value, the models have achieved lower ECE, indicating that the neural network has good calibration. The output of the proposed model can match its confidence.

By comprehensive analysis, the proposed method has certain advantages in two datasets, which verifies that it is stable and generalizable. Since this work proposes a global 3D reconstruction, it can have a more complete global digital information than 2D segmentation and local patch segmentation. In the existing mainstream 3D network architecture, 3D U-Net is a lightweight and cost-effective network. Combined with data enhancement and loss function proposed in this work, it has certain clinical potential.

### 4.3. Limitations and Outlook

There are also some limitations to this study. Based on the proposed weighted loss function, the parameter ratios discussed are not absolute, and the optimal results may change under other datasets. Therefore, in the next stage, a good idea is to change the α and γ parameters to be learnable. We believe it will be able to adapt to more application areas. Therefore, this work can establish a quantitative model for the infection in COVID-19 CT images, which is helpful to assist the use of computers in the next stage to complete the needs of intelligent diagnosis. In addition, the proposed network model and method is a universal method, which can be extended to other application fields, especially other respiratory diseases. We will seek the law of various lung CT images in the next task, not limited to COVID-19.

## 5. Conclusions

This work proposed a method for extracting infection from COVID-19 CT images. It mainly included lung region extraction, data enhancement, and convolutional neural network segmentation. In the process of lung region extraction, a Random Walk strategy based on threshold and spatial location can distinguish complicated lung boundary problems caused by infection; In addition, through inhibiting structures such as vessels and tracheas, it can reduce interference of tissues in lungs; Finally, a new network DW-UNet based on weighted loss function was proposed, which can balance the cost of all samples, making the training process of convolutional neural networks more reliable.

The proposed loss function can solve actual situation of imbalance between positive and negative samples, which will be applied to extraction of small tissues such as infection, nodules, and vessels in medical images. When facing different applications, results can be more reliable by adjusting parameters and selecting appropriate network, which also provides a new direction for convolutional neural network processing of medical images. Notably, the proposed data enhancement is dominated by vessels and tracheas throughout lung, but it may also cause partially spreading filamentous infection to be misjudged as vascular tissue. Therefore, how to distinguish filamentous spread of blood vessels, trachea, and infection provides a direction for further research.

## Figures and Tables

**Table 1:** Analysis of the Pros and Cons of Related Research.

Methods	Advantages	Disadvantages
Fan et al. [21]	The training framework on semi-supervised learning can cope with the shortage of labeled data.	The network is only used for the processing of two-dimensional slices. Therefore, it is necessary to manually screen slices with infected areas from CT images in advance.
Qiu et al. [22]	The model is lightweight and can adapt to deployment and computing requirements.	Due to insufficient network information extraction, some segmentation results lead to loss of details.
Yan et al. [23]	The design of pyramid space enables the network to integrate information from multiple stages in the learning process. It has a stronger learning ability.	There are many network parameters, the training speed is slow, and the hardware requirements are high.
Chen et al. [24]	The location of the infection and the 3D attention module can improve the texture characteristics of the target area.	The network is only used for partial segmentation, and cannot display global spatial information.

**Table 2:** Comparison of the segmentation with or without data enhancement (compared with the manual ground truth).

	ACC (%)	PRE (%)	REC (%)	DSC (%)	SPE (%)	MCC (%)	Kappa (%)
True *	99.94 ± 00.01	58.21 ± 15.75	67.25 ± 13.51	60.11 ± 13.93	99.96 ± 0.01	61.35 ± 13.21	60.09 ± 13.93
False *	99.94 ± 00.02	73.42 ± 13.35	26.86 ± 11.79	37.50 ± 14.57	99.99 ± 0.00	42.66 ± 13.53	37.49 ± 14.60

* True represents output with data enhancement. False is outputs without data enhancement.

**Table 3:** Chi-square tests for data augmentation.

	Value	Exact Significance (2-Sided)
McNemar Test		0.000 *
N of Valid Cases	2048917488	

* Binomial distribution used.

**Table 4:** ACC, PRE, REC, and DSC comparison of the segmentation with different parameters on focal loss function (compared with the manual ground truth).

$L_{f}\left(\alpha ,\gamma \right)$	ACC (%)	PRE (%)	REC (%)	DSC (%)
(0.8, 2)	99.94 ± 00.02	57.29 ± 14.30	76.09 ± 11.27	62.17 ± 11.03
(0.6, 2)	99.93 ± 00.02	53.20 ± 14.28	64.29 ± 10.70	56.32 ± 11.83
(0.4, 2)	99.94 ± 00.02	70.80 ± 11.84	49.12 ± 11.50	56.75 ± 09.50
(0.2, 2)	99.94 ± 00.01	67.55 ± 15.27	51.22 ± 11.25	56.35 ± 11.72
(0.8, 1)	99.94 ± 00.02	60.55 ± 14.63	65.98 ± 12.03	60.50 ± 10.56
(0.6, 1)	99.89 ± 00.02	40.25 ± 13.10	73.70 ± 08.95	49.30 ± 12.83
(0.4, 1)	99.95 ± 00.01	68.22 ± 15.72	55.63 ± 13.12	58.87 ± 12.40
(0.2, 1)	99.94 ± 00.02	68.22 ± 15.59	51.43 ± 12.63	56.96 ± 11.66

**Table 5:** ACC, PRE, REC, and DSC comparison of the segmentation with different parameters on weighted loss function (compared with the manual ground truth).

$L_{w}\left(\alpha ,\gamma \right)$	ACC (%)	PRE (%)	REC (%)	DSC (%)
(0.8, 2)	99.88 ± 00.03	40.46 ± 13.02	84.64 ± 06.58	52.08 ± 13.35
(0.6, 2)	99.88 ± 00.03	40.31 ± 13.35	81.82 ± 07.69	51.18 ± 13.73
(0.4, 2)	99.93 ± 00.02	51.54 ± 07.57	74.34 ± 10.72	58.23 ± 13.56
(0.2, 2)	99.94 ± 00.02	64.16 ± 15.13	56.97 ± 11.11	58.24 ± 10.53
(0.8, 1)	99.90 ± 00.03	45.58 ± 14.12	82.56 ± 07.56	55.61 ± 13.38
(0.6, 1)	99.91 ± 00.03	49.14 ± 14.70	79.89 ± 08.10	57.46 ± 12.54
(0.4, 1)	99.93 ± 00.02	54.78 ± 15.31	74.25 ± 11.75	60.18 ± 12.40
(0.2, 1)	99.94 ± 00.02	60.42 ± 12.58	70.79 ± 10.45	63.15 ± 09.33

**Table 6:** ACC, PRE, REC, and DSC comparison of the segmentation with different loss function (compared with the manual ground truth).

Methods	ACC (%)	PRE (%)	REC (%)	DSC (%)
$L_{bce}$	99.94 ± 00.01	58.21 ± 15.75	67.25 ± 13.51	60.11 ± 13.93
$L_{f}\left(0.8,2\right)$	99.94 ± 00.02	57.29 ± 14.30	76.09 ± 11.27	62.17 ± 11.03
$L_{f}\left(0.8,1\right)$	99.94 ± 00.02	60.55 ± 14.63	65.98 ± 12.03	60.50 ± 10.56
$L_{w}\left(0.8,2\right)$	99.88 ± 00.03	40.46 ± 13.02	84.64 ± 06.58	52.08 ± 13.35
$L_{w}\left(0.8,1\right)$	99.90 ± 00.03	45.58 ± 14.12	82.56 ± 07.56	55.61 ± 13.38
$L_{w}\left(0.2,1\right)$	99.94 ± 00.02	60.42 ± 12.58	70.79 ± 10.45	63.15 ± 09.33

**Table 7:** Comparison of the segmentation with different methods on private dataset (compared with the manual ground truth).

Methods	ACC (%)	PRE (%)	REC (%)	DSC (%)	MCC (%)	Kappa (%)	ECE (%)
V-Net	99.94 ± 00.01	67.75 ± 19.47	44.22 ± 13.24	51.15 ± 14.40	53.38 ± 14.10	51.14 ± 14.40	00.14 ± 00.03
U-Net	99.94 ± 00.01	58.21 ± 15.75	67.25 ± 13.51	60.11 ± 13.93	61.35 ± 13.21	60.09 ± 13.93	00.24 ± 00.03
Dense-Net	99.94 ± 00.02	74.86 ± 15.81	40.62 ± 14.77	49.50 ± 13.80	53.25 ± 12.75	49.49 ± 13.80	00.05 ± 00.01
DenseVoxel-Net	99.67 ± 00.19	25.16 ± 12.06	88.44 ± 05.70	36.08 ± 14.91	43.59 ± 12.51	36.05 ± 14.90	01.34 ± 00.31
Our	99.94 ± 00.02	60.42 ± 12.58	70.79 ± 10.45	63.15 ± 09.33	64.33 ± 08.79	63.14 ± 09.33	00.30 ± 00.02

**Table 8:** Chi-square tests for different models.

	Value	Degree of Freedom	Asymptotic Significance (2-Sided)
Pearson Chi-Square	188,564,555.194 *	4	0.000
Likelihood Ratio	190,509,995.678	4	0.000
Linear-by-Linear Association	64,355.139	1	0.000
N of Valid Cases	5,122,293,720		

* 0 cells (0.0%) have expected count less than 5. The minimum expected count is 4.91 × 10^8^.

**Table 9:** ACC, PRE, REC, and DSC comparison of the segmentation with different methods on public dataset (compared with the manual ground truth).

Methods	ACC (%)	PRE (%)	REC (%)	DSC (%)
V-Net	99.66 ± 00.15	81.66 ± 05.22	15.90 ± 06.10	25.53 ± 08.77
U-Net	99.71 ± 00.12	65.34 ± 09.08	44.71 ± 09.31	51.70 ± 08.28
Dense-Net	99.69 ± 00.14	87.47 ± 03.89	22.74 ± 07.15	34.98 ± 09.06
DenseVoxel-Net	99.19 ± 00.21	29.17 ± 09.56	80.81 ± 05.18	41.13 ± 10.51
Our	99.73 ± 00.12	77.02 ± 06.06	41.23 ± 08.61	52.50 ± 08.18

## Data Availability

The datasets generated during this study are available from the corresponding author on reasonable request.

## Supplementary Materials

The following are available online at https://www.mdpi.com/article/10.3390/diagnostics11111942/s1, Table S1: Details of each subject in the private dataset. Table S2: Details of each subject in the public dataset. Table S3: Var. 1 * Var. 2 Crosstabulation for Data Augmentation. Table S4: Var.1 * Var.2 Crosstabulation for different models.
