# Supplementary material for: DW-UNet: Loss Balance under Local-Patch for 3D Infection Segmentation from COVID-19 CT Images
Source: Diagnostics (Basel). 2021 Oct 20;11(11):1942. doi: 10.3390/diagnostics11111942 (PMC8623821; doi:10.3390/diagnostics11111942)
Supplement: Supplementary file 1 [file diagnostics-11-01942-s001.zip › diagnostics-1362049-supplementary.pdf]

diagnostics

MDPI

Supporting information to: "DW-UNet: Loss Balance Under Local-Patch for 3D Infection Segmentation from COVID-19 CT Images"

Cheng Chen [1,&], Jiancang Zhou [2,&], Kangneng Zhou [1], Zhiliang Wang [1] and Ruoxiu Xiao [1,*]

1 School of Computer and Communication Engineering, University of Science and Technology Beijing, Beijing 100083, China; xiaoruoxiu@ustb.edu.cn
2 Department of critical care medicine, Sir Run Run Shaw hospital, Zhejiang University school of medicine, Hangzhou, 310016, China; jiancangzhou@zju.edu.cn
* Correspondence: xiaoruoxiu@ustb.edu.cn; Tel.: Tel.: +86-15210326132
& Cheng Chen and Jiancang Zhou contributed to this work equally.

This supporting information is used to supplement the main text. During the evaluation process, this work selected two datasets to test the proposed method. The CT images of 20 of the cases came from the Fifth Medical Center of the PLA General Hospital. The details of each subject are reported in Table SI. Another data set is selected from Kaggle's public COVID-19 data set, and its details are reported in Table SII.

Table S1 Details of each subject in the private dataset

Subject ID	Number of slices	Allocation	Subject ID	Number of slices	Allocation
Subject 01	529	Training set	Subject 11	489	Training set
Subject 02	529	Training set	Subject 12	553	Training set
Subject 03	569	Training set	Subject 13	537	Testing set
Subject 04	553	Training set	Subject 14	593	Testing set
Subject 05	521	Training set	Subject 15	529	Testing set
Subject 06	513	Training set	Subject 16	513	Testing set
Subject 07	553	Training set	Subject 17	561	Testing set
Subject 08	529	Training set	Subject 18	245	Testing set
Subject 09	513	Training set	Subject 19	489	Testing set
Subject 10	473	Training set	Subject 20	441	Testing set

Table S2 Details of each subject in the public dataset [1][2]

Subject ID	Number of slices	Allocation
Subject 01	301	Testing set
Subject 02	200	Testing set
Subject 03	290	Testing set
Subject 04	213	Testing set
Subject 05	249	Testing set
Subject 06	301	Testing set
Subject 07	256	Testing set
Subject 08	301	Testing set

This study analyzed the results of data augmentation and different models through statistical tests to ensure that there are significant differences between the different results. Regarding the McNemar test [3] used for data augmentation in the manuscript, the Var. 1 * Var. 2 crosstabulation is shown in Table S3. Also, the Var. 1 * Var. 2 crosstabulation for different models is presented in Table S4.

Table S3 Var. 1 * Var. 2 Crosstabulation for Data Augmentation

			Var.2		Total
			0	1	
Var.1	0	Count	656415613	368043131	1024458744
		Expected Count	531485132.0	492973612.0	1024458744.0
		% within Var.1	64.1%	35.9%	100.0%
		% within Var.2	61.8%	37.3%	50.0%
	1	Count	406554651	617904093	1024458744
		Expected Count	531485132.0	492973612.0	1024458744.0
		% within Var.1	39.7%	60.3%	100.0%
		% within Var.2	38.2%	62.7%	50.0%
Total		Count	1062970264	985947224	2048917488
		Expected Count	1062970264.0	985947224.0	2048917488.0
		% within Var.1	51.9%	48.1%	100.0%
		% within Var.2	100.0%	100.0%	100.0%

Table S4 Var.1 * Var.2 Crosstabulation for different models

			Var.2		Total
			.00	1.00	
Var.1	.00	Count	496501995	527956749	1024458744
		Expected Count	490774556.4	533684187.6	1024458744.0
		% within Var.1	48.5%	51.5%	100.0%
		% within Var.2	20.2%	19.8%	20.0%
	1.00	Count	406554651	617904093	1024458744
		Expected Count	490774556.4	533684187.6	1024458744.0
		% within Var.1	39.7%	60.3%	100.0%
		% within Var.2	16.6%	23.2%	20.0%
	2.00	Count	516096268	508362476	1024458744
		Expected Count	490774556.4	533684187.6	1024458744.0
		% within Var.1	50.4%	49.6%	100.0%
		% within Var.2	21.0%	19.1%	20.0%
	3.00	Count	657054022	367404722	1024458744
		Expected Count	490774556.4	533684187.6	1024458744.0
		% within Var.1	64.1%	35.9%	100.0%
		% within Var.2	26.8%	13.8%	20.0%
	4.00	Count	377665846	646792898	1024458744
		Expected Count	490774556.4	533684187.6	1024458744.0
		% within Var.1	36.9%	63.1%	100.0%
		% within Var.2	15.4%	24.2%	20.0%
Total		Count	2453872782	2668420938	5122293720
		Expected Count	2453872782.0	2668420938.0	5122293720.0
		% within Var.1	47.9%	52.1%	100.0%
		% within Var.2	100.0%	100.0%	100.0%

Reference

1. Coronacases.org, “Coronavirus Cases,” [Online]. Available: https://coronacases.org/.
2. Radiopaedia, “COVID-19 Pneumonia,” [Online]. Available: https://radiopaedia.org/playlists/25887.
3. Performance Assessment. In Statistical Pattern Recognition; 2011; pp. 404-432.
